# Prognostic significance of N-Terminal Pro-BNP in patients with COVID-19 pneumonia without previous history of heart failure

**DOI:** 10.34172/jcvtr.2021.26

**Published:** 2021-04-24

**Authors:** Murat Selçuk, Muhammed Keskin, Tufan Çınar, Nuran Günay, Selami Doğan, Vedat Çiçek, Şahhan Kılıç, Süha Asal, Samet Yavuz, Nurgül Keser, Ahmet L. Orhan

**Affiliations:** ^1^Cardiology, Health Sciences University, Sultan II. Abdulhamid Han Training and Research Hospital, Istanbul, Turkey; ^2^Cardiology, Health Sciences University, Umraniye Training and Research Hospital, Istanbul, Turkey

**Keywords:** COVID-19, NT-proBNP, In-Hospital Mortality, Heart Failure

## Abstract

***Introduction:*** The objective of the present research was to evaluate the possible association between the N-terminal pro-brain type natriuretic peptide (NT-proBNP) levels and in-hospital mortality in coronavirus disease 2019 (COVID-19) pneumonia patients who did not have pre-existing heart failure (HF).

***Methods:*** A total of 137 consecutive patients without pre-existing HF and hospitalized due to COVID-19 pneumonia were enrolled into the current research. The main outcome of the research was the in-hospital death. The independent parameters linked with the in-hospital death were determined by multivariable analysis.

***Results:*** A total of 26 deaths with an in-hospital mortality rate of 18.9% was noted. Those who died were older with an increased frequency of co-morbidities such as hypertension, chronic kidney disease, coronary artery disease, stroke and dementia. They had also increased white blood cell (WBC) counts and had elevated glucose, creatinine, troponin I, and NT-pro-BNP levels but had decreased levels of hemoglobin. By multivariable analysis; age, NT-pro-BNP, WBC, troponin I, and creatinine levels were independently linked with the in-hospital mortality. After ROC evaluation, the ideal value of the NT-pro-BNP to predict the in-hospital mortality was found as 260 ng/L reflecting a sensitivity of 82% and a specificity of 93% (AUC:0.86; 95%CI:0.76-0.97).

***Conclusion:*** The current research clearly shows that the NT-proBNP levels are independently linked with the in-hospital mortality rates in subjects with COVID-19 pneumonia and without HF. Thus, we believe that this biomarker can be used as a valuable prognostic parameter in such cases.

## Introduction

Coronavirus disease 2019 (COVID-19) is characterized by pulmonary failure due to severe acute respiratory disease syndrome and is usually complicated with multisystem involvement.^1^ The first stage of respiratory involvement may be followed by a severe host inflammatory response syndrome. Cardiac involvement is one of the inflammatory response syndromes, which is linked with a poor prognosis. Furthermore, COVID-19 subjects with an accompanying cardiovascular disease (CVD) have been found to have a higher rate of unfavorable outcomes when compared to others.^2^ However, the possible association between COVID-19 infection and the cardiovascular system has still not been completely analyzed. Acute myocarditis is a frequent result of cardiac involvement, where the diagnosis is usually done by cardiac troponin measurements.^2^ There are other parameters, such as creatine kinase myocardial band, neutrophil-to-lymphocyte ratio, and lactate dehydrogenase, all of which were postulated as the predictors of death in COVID-19 disease.^3-5^

The measurement of plasma natriuretic peptides (NPs) is commonly considered as one of the initial diagnostic tools in acute heart failure (HF) patients. Brain type NPs and N-terminal pro-BNP (NT-proBNP) are the most common forms of measured NPs.^6-8^ Since NT-proBNP levels may also elevate in pneumonia, the prognostic importance of this biomarker has been evaluated in COVID-19 subjects in various studies. In a relatively small study enrolling only 54 COVID-19 cases, Gao et al. found that NT-proBNP levels were independently related with in-hospital mortality in severe COVID-19 cases.^9^ Moreover, Calvo-Fernández measured NT-proBNP levels in 506 COVID-19 patients and found that NT-proBNP levels were independently related with death or mechanical ventilation in such kind of patients.^10^ However, COVID-19 patients with HF were not excluded in all of these studies despite the fact that the elevated NT-proBNP levels might have also resulted from an acute HF decompensation. We noticed that the prognostic value of increased NT-proBNP levels have not been comprehensively analyzed in COVID-19 patients who were free from HF although there is a high probability of cardiac injury in such cases. Thus, in this retrospective and observational study, we tried to address this issue and evaluated the prognostic value of NT-proBNP levels on in-hospital outcomes in subjects without HF and hospitalized due to COVID-19 pneumonia.

## Material and methods

### Study cohort

We performed a retrospective cohort research using the data from consecutive COVID-19 cases admitted to pandemic center. Patients with HF who were diagnosed either during initial evaluation with physical examination or reported by the history given by the patient were excluded from the study. In addition, we scanned all electronic health records of all cases with the ICD code I50 to confirm or exclude the diagnosis of HF. Patients either with end-stage renal disease requiring dialysis or with acute hepatic failure were also excluded and thus, totally 137 COVID-19 cases were enrolled into the study. The data, including demographic features and laboratory results, were explicitly obtained from the hospital electronic medical database.The diagnosis of COVID 19 disease was done by RT-PCR test and all cases had typical COVID-19 signs and symptoms as well as typical pulmonary imaging findings. According to the National Health Ministry COVID-19 treatment recommendations, patients were treated either with hydroxychloroquine or azithromycin as for the first line treatment protocol. As the second line treatment option, either favipiravir or remdesivir was given upon a failure of first-line approach. In case of a severe cytokine storm, corticosteroids and biologic agents were applied.

### Laboratory evaluation

Following admission to the emergency department, blood samples for standard hematology and biochemical parameters, including cardiac troponin and NT-pro-BNP levels, were obtained from all subjects. NT-pro-BNP level was determined by the chemiluminescence (CL) method using I2000 architect machine (Abbott, USA). The normal range of NT-pro-BNP in our institution was reported as 0–300 ng/L. Standard hematology and biochemical parameters were measured using a Beckman Coulter LH 780 machine (Beckman Coulter, USA).

### Primary outcome

The primary outcome of the research was the in-hospital death and the in-hospital death was defined as a death that occurred during index hospitalization.

### Statistical analysis

All statistical analyses were conducted using the SPSS software programme (Version 23.0, SPSS, USA). To test the normality of the database, we used the Shapiro-Wilk’s test. All categorical parameters were displayed as numbers and percentages. The categorical parameters were assessed and compared using the Chi-square test. Continuous parameters with normal distribution were analyzed using the independent sample *t*test. For continuous parameters without normal distribution, we used the Mann–Whitney* U* test for comparison. The independent parameters linked with in-hospital mortality were determined using both univariate and multivariable analyses. The cut-point for selection of the covariates from the univariate analysis to be included into the multivariable analysis was a *P* value < 0.10. A receiver operating characteristic (ROC) examination was done to determine the ideal value of NT-pro-BNP in predicting the in-hospital mortality. In order to determine the cumulative survival of COVID-19 cases along with the ideal value of NT-pro-BNP, we used the Kaplan–Meir curve analysis. All results were evaluated with a statistical significance level of *P* value < 0.05.

## Results

Totally, 137 COVID-19 cases met the inclusion criteria of the research and 52.5% of the patients (n=72 patients) were male. The research cohort was classified into two groups; group I; with patients who died and group II; patients who survived during the index hospitalization. We observed a total of 26 deaths resulting in an in-hospital mortality rate of 18.9%. Demographic features and laboratory data are demonstrated in Table 1. Those cases in the group I were older and the co-morbidities, including hypertension, chronic kidney disease, coronary artery disease, stroke and dementia, were more commonly observed. There was no difference between the two groups in regard to other demographic features. In terms of previous treatments, the use of aspirin, P_2_Y_12_ inhibitors, statins, angiotensin receptor blockers, calcium channel blockers, and beta blockers were more frequent in cases who died. As for the laboratory findings, those in the group I were found to have increased levels of white blood cell (WBC) count and glucose, creatinine, troponin I and NT-pro-BNP levels and decreased levels of hemoglobin. Other laboratory data were not different between the groups.

**Table 1:** Characteristics of survivor and non-survivor patients hospitalized with the diagnosis of COVID-19 pneumonia

**Characteristics**	**Survivor** **(n=111)**	**Non-Survivor** **(n=26)**	***P*** ** value**
Age	55 ± 14	66 ± 14	<0.001
Gender			
Male	55 (49.5)	17 (65.4)	0.146
Comorbidities, n (%)			
Hypertension	45 (40.5)	18 (69.2)	0.008
Diabetes mellitus	27 (24.3)	9 (34.6)	0.283
Hyperlipidemia	14 (12.6)	3 (11.5)	0.881
Smoking	8 (7.2)	1 (3.8)	0.534
Chronic lung disease	14 (12.6)	6 (23.1)	0.174
Chronic kidney disease	6 (5.4)	6 (23.1)	0.004
Coronary artery disease	8 (7.2)	11 (42.3)	<0.001
Stroke	1 (0.9)	2 (7.7)	0.033
Dementia	0 (0.0)	2 (7.7)	0.003
Cancer	3 (2.7)	2 (7.7)	0.222
Previous medications, n (%)			
Aspirin	15 (13.5)	11 (42.3)	<0.001
P_2_Y_12_ inhibitors	5 (4.5)	6 (23.1)	0.002
Anticoagulant therapy	3 (2.7)	2 (7.7)	0.222
Statins	8 (7.2)	8 (30.8)	0.001
Angiotensin receptor blockers	25 (22.5)	20 (76.9)	<0.001
Calcium channel blockers	13 (11.7)	7 (26.9)	0.048
Beta blockers	14 (12.6)	9 (34.6)	0.007
Insulin	4 (3.6)	2 (7.7)	0.359
Oral anti-hyperglycemic agents	17 (15.3)	7 (26.9)	0.161
Laboratory data			
White blood cell count, cells/µL	6.2 ± 2.7	9.9 ± 4.9	<0.001
Platelets, cells/µL	217 ± 72	233 ± 75	0.315
Hemoglobin, g/dL	12.7 ± 1.7	11.4 ± 3.0	0.003
Glucose, mg/dL	115 ± 46	162 ± 102	<0.001
C-reactive protein, mg/dL	57 ± 63	40 ± 54	0.245
Creatinine, mg/dL	0.97 ± 0.25	1.49 ± 0.91	<0.001
N-terminal pro-BNP, ng/L	104 ± 140	845 ± 573	<0.001
Troponin I, ng/L	53 ± 142	593 ± 1739	<0.001
Hospital stays, d	8 ± 4	10 ± 8	0.098

**Figure 1:**
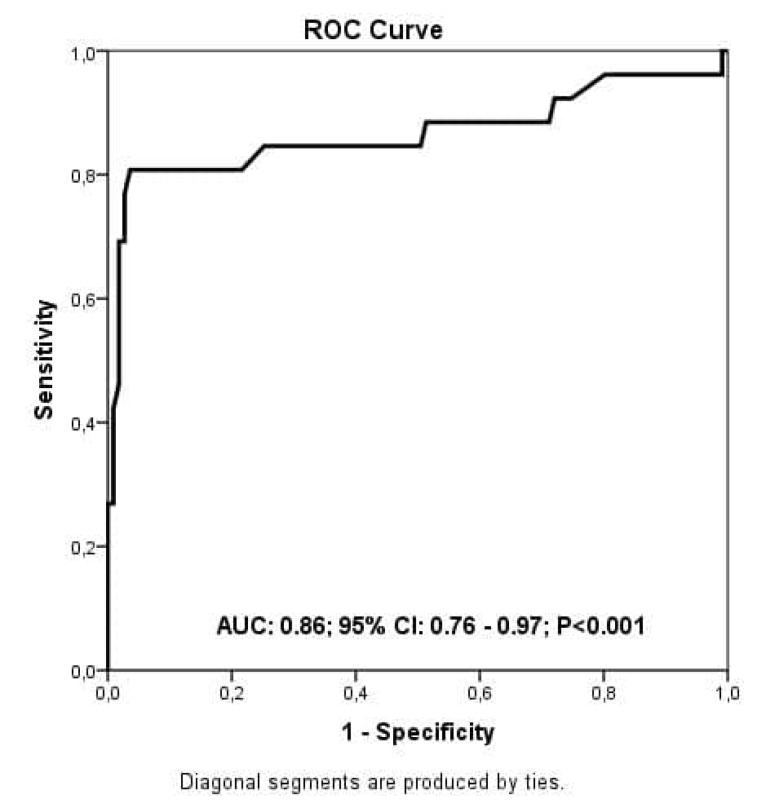
The ideal value of NT-pro-BNP in predicting the in-hospital mortality in COVID-19 cases without HF

**Figure 2:**
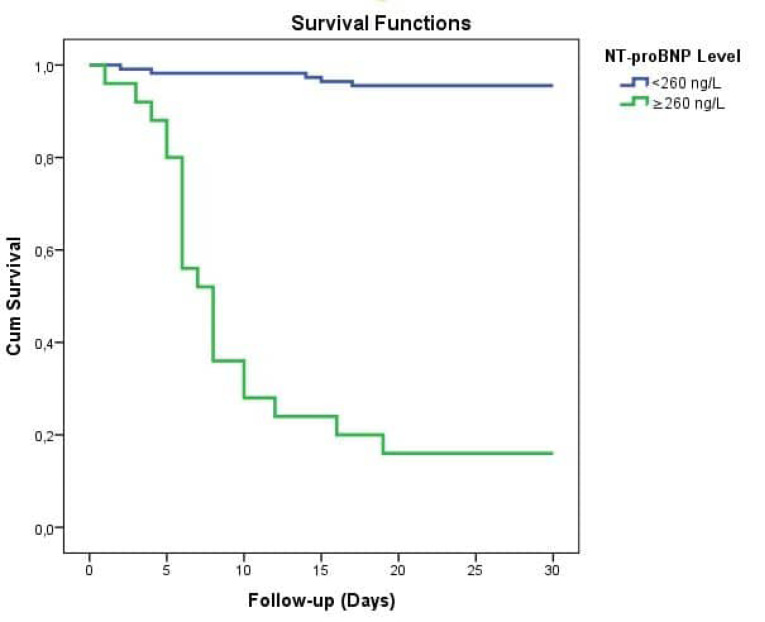
In a Kaplan-Meier survival curve analysis, COVID-19 cases with NT-pro-BNP levels higher than 260 ng/L had higher incidence of in-hospital deaths compared to those with lower NT-pro-BNP levels

The independent parameters linked with the in-hospital mortality were determined by both univariate and multivariable analysis. With the univariate analysis, a total of ten parameters were found to be linked with the in-hospital mortality. Those parameters were age, accompanying hypertension, chronic kidney disease and coronary artery disease, WBC count, NT-Pro-BNP (per 100 ng/L), creatinine, troponin I and plasma glucose levels. As shown in Table 2, multivariable analysis revealed that the independent parameters linked with the in-hospital mortality were age, WBC, creatinine, troponin I and NT-Pro-BNP (per 100 ng/L) (OR: 1.64.5; 95%CI: 1.38–2.06; *P* = 0.001). In a ROC examination, the ideal value of the NT-pro-BNP in predicting the in-hospital mortality was found as 260 ng/L reflecting an 82% sensitivity and an 93% specificity (AUC: 0.86; 95% CI: 0.76-0.97) (Figure 1). Kaplan-Meir survival curve analysis revealed that COVID-19 cases with NT-pro-BNP levels > 260 ng/L had higher in-hospital death rates when compared to those with lower NT-pro-BNP levels [Log rank test, *P* value <0.001] (Figure 2).

**Table 2:** Univariate analysis and multivariable model for in-hospital mortality^a^

**Univariate analysis**	***P *** **value**	**OR (95% CI)**	**Multivariate analysis**	
			***P*** ** value**	**OR (95% CI)**
Age	<0.001	1.06 (1.02 – 1.10)	0.001	1.06 (1.02 – 1.10)
Male gender	0.150	1.92 (0.79 – 4.68)	**-**	**-**
Hypertension	0.011	3.30 (1.32 – 8.23)	**-**	**-**
Diabetes mellitus	0.286	1.64 (0.65 – 4.12)	**-**	**-**
Chronic lung disease	0.180	2.07 (0.71 – 6.06)	**-**	**-**
Chronic kidney disease	0.030	3.30 (1.12 – 9.72)	**-**	**-**
Coronary artery disease	<0.001	6.16 (2.68 –14.13)	**-**	**-**
Smoking	0.540	0.51 (0.06 – 4.30)	**-**	**-**
NT-Pro-BNP (per 100 ng/L)	<0.001	1.75 (1.41 – 2.18)	<0.001	1.64 (1.38 – 2.06)
Troponin I	<0.001	1.00 (1.00 – 1.00)	<0.001	1.00 (1.00 – 1.00)
C-reactive protein	0.208	0.99 (0.99 – 1.00)	-	-
White blood cell	<0.001	1.28 (1.13 – 1.44)	0.032	1.44 (1.03 – 1.98)
Hemoglobin	0.013	0.76 (0.62 – 0.94)	-	--
Platelet	0.314	1.00 (0.99 – 1.00)	-	-
Creatinine	<0.001	22.80 (4.69 – 110.69)	0.021	7.89 (1.25 – 31.24)
Glucose	<0.001	1.01 (1.00 – 1.01)	-	-

Abbreviations: CI, confidence interval; OR, Odds ratio ^a^All clinically relevant parameters were included in the model.

## Discussion

Our study has several key findings; (I) NT-proBNP levels are independently linked with in-hospital mortality in COVID-19 pneumonia subjects without HF, (II) NT-proBNP can be utilized as a prognostic biomarker in subjects hospitalized with COVID-19 pneumonia who were free from HF.

Since the beginning of the pandemic in December 2019, there has been a vastly accumulating evidence that COVID-19 subjects with preexisting cardiovascular disease (CVD) are more vulnerable to the infection and prone to unfavorable outcomes.^1^ However, patients with COVID-19 without a prior documented CVD were also found to have suffered from an acute cardiovascular event.^2^ In the current literature, there is a growing evidence demonstrating a high rate of myocardial injury in COVID-19 patients.^1,11^ Therefore, in all patients admitted to our center, cardiac troponin I and/or NT-proBNP measurements were performed for determining a possible myocardial injury.

An elevated NT-proBNP level, which is accepted as a usual marker for HF diagnosis, may be linked with poor outcomes in acute respiratory dysfunction syndrome (ARDS) subjects.^12^ Moreover, a recent retrospective study suggested a possible direct cardiac injury in COVID-19 patients linking elevated levels of NT-proBNP levels to mortality.^13^ Therefore, this biomarker can also be utilized as a useful indicator for the severity of COVID-19 infection. Several mechanisms are blamed for the elevation of NT-proBNP in COVID-19 cases. First, the use of a vasopressor therapy as well as hypoxia-induced pulmonary vasocontraction can lead to the increasing levels of NT-proBNP.^12^ Second, the NT-proBNP release can also be triggered by direct involvement of the myocardium tissue by the activation of the inflammatory process, demand-supply mismatch, and oxidative stress.^13^ Finally, the development of acute renal failure which, by impairing its clearance, may also elevate the levels of NT-proBNP.^12,13^

The prognostic power of NT-proBNP levels to predict the in-hospital mortality was investigated in prior studies.^9,10^ These studies reported that the elevated NT-proBNP levels were linked with higher mortality rates in COVID-19 cases. Furthermore, a recent meta-analysis indicated that evaluating NT-proBNP can aid physicians to discriminate COVID-19 patients at high risk.^14^ However, another meta-analysis, reported by Dawson et al., has not reported a significant dissimilarities in NT-proBNP levels between the patients who died or were critically ill with those who survived or were not critically ill.^5^ The main reason of the similarity of NT-proBNP levels in both groups in this meta-analysis was possibly due to the inclusion of patients with pre-existing HF. However, in our study, we paid special attention to keep the study cohort homogeneous. We only enrolled COVID-19 cases without HF for better evaluation of the prognostic role of NT-proBNP in such patients. We conclude that evaluating the NT-proBNP level provides crucial information in COVID-19 patients with pneumonia who are free from HF. Interestingly, we find that the threshold used for NT-proBNP in this study is lower than the cut-points used in clinic and clinical trials for diagnosis of HF (<300 ng/L or < 400 ng/L less likely to be HF). This finding may imply that elevated NT-proBNP levels, even within the upper limit of normal reference range, could indicate an occult cardiac injury in COVID-19 cases. Finally, an elevated NT-proBNP level, in particular, suggests that clinicians should carry out a more precise cardiac examination to exclude a direct or indirect myocardial involvement in such patients.

Several limitations of our research should be noted. First, this was an observational and retrospective research. Second, there might have been other possible confounders of in-hospital death, which could have been omitted. Third, despite the fact that we performed a detailed physical examination and scanned electronic health records of all cases with the ICD code I50 to confirm the diagnosis of HF, there might have been some cases with undocumented HF. In addition, many patients had a history of hypertension and coronary artery disease; thus, some might have HF, particularly HF with preserved left ventricle ejection fraction. Fourth, D-dimer levels were not evaluated during the initial evaluation in our study. Fifth, in spite of having a limited specificity and sensitivity for HF diagnosis, the evaluation of the baseline electrocardiography could have been included in the study. Sixth, our initial management protocols may not be completely compatible with the current recommendations. Finally, due to the relatively small patient population, a study with a larger patient cohort with similar enrollment criteria is warranted to confirm our study findings.

## Conclusion

The current study shows that NT-proBNP might be considered as a prognostic indicator of in-hospital death in COVID-19 cases with pneumonia who were free from a pre-existing HF.

## Competing interest

There is no conflict of interest to be mentioned by the authors.

## Ethical approval

The study protocol complies with the Declaration of Helsinki and approved by both the Ethics Committee and Ministry of Health (approval number: B.10.1.TKH.4.34.H.GP.0.01/149). There was no need for an informed consent owing to the design of the research.

## Funding

None.
